# Measuring the Uncertainty of Predictions in Deep Neural Networks with Variational Inference

**DOI:** 10.3390/s20216011

**Published:** 2020-10-23

**Authors:** Jan Steinbrener, Konstantin Posch, Jürgen Pilz

**Affiliations:** 1 Control of Networked Systems Group, Department of Smart Systems Technologies, Universität Klagenfurt, Universitätsstr 65-67, 9020 Klagenfurt, Austria; 2 CTR Carinthian Tech Research AG, Europastr 12, 9524 Villach, Austria; 3 Department of Statistics, Universität Klagenfurt, Universitätsstr 65-67, 9020 Klagenfurt, Austria; konstantin.posch@aau.at (K.P.); juergen.pilz@aau.at (J.P.)

**Keywords:** Bayesian deep learning, model uncertainty, variational inference, image classification

## Abstract

We present a novel approach for training deep neural networks in a Bayesian way. Compared to other Bayesian deep learning formulations, our approach allows for quantifying the uncertainty in model parameters while only adding very few additional parameters to be optimized. The proposed approach uses variational inference to approximate the intractable a posteriori distribution on basis of a normal prior. By representing the a posteriori uncertainty of the network parameters per network layer and depending on the estimated parameter expectation values, only very few additional parameters need to be optimized compared to a non-Bayesian network. We compare our approach to classical deep learning, Bernoulli dropout and Bayes by Backprop using the MNIST dataset. Compared to classical deep learning, the test error is reduced by 15%. We also show that the uncertainty information obtained can be used to calculate credible intervals for the network prediction and to optimize network architecture for the dataset at hand. To illustrate that our approach also scales to large networks and input vector sizes, we apply it to the GoogLeNet architecture on a custom dataset, achieving an average accuracy of 0.92. Using 95% credible intervals, all but one wrong classification result can be detected.

## 1. Introduction

Deep learning has led to series of breakthroughs in many fields of applied machine learning, especially in image classification [1] or natural language processing [2]. In 1989, the universal approximation theorem was proven, which can be summarized that a feed-forward network with one hidden layer can approximate a broad class of functions arbitrarily well [3]. Currently, it has been shown that for a given bound of the approximation error, deep networks require exponentially less data than shallow ones [4]. The possible applications of deep neural networks for classification and detection cover a wide range including medical imaging, psychology, automotive, industry, finance and life sciences [5,6,7,8,9,10].

Despite its potential and superior accuracy for classification tasks compared to other techniques, dissemination of deep learning into real-world applications and services has been limited by a lack of information about model uncertainty (epistemic uncertainty, parameter uncertainty), see Reference [11]. Indeed prediction uncertainty decomposes into epistemic and aleatoric uncertainty. Aleatoric uncertainty captures noise inherent in the observations and is covered by the distribution used to define the likelihood function. Thus, aleatoric uncertainty is also considered in frequentist deep learning. However, standard deep networks for classification and regression do not represent model uncertainty since network parameters are considered to be deterministic values. Consequently, the overall prediction uncertainty information is of limited use. How can one be confident about a fixed parameter specification in a highly parameterized deep learning model?  Gal and Ghahramani [12] have shown that a frequentist classification network can guess randomly while returning a high class probability. The rather unreliable uncertainty information returned by classical deep nets affects those applications, where wrong decisions based on false classification results could have significant negative or even catastrophic impact such as in self-driving cars, finance or medical applications [13]. Often it is essential to know how sure a network is about a special prediction and not only that it predicts on average quite well.

Besides the inability of classical deep nets to represent model uncertainty they are prone to overfitting. Modern deep models cover a huge amount of parameters and therefore require a huge amount of labeled training data as well. In many applications, such an amount cannot be provided because of financial or time constraints. To overcome this problem, the deep learning community introduced several probabilistic regularization techniques, such as dropout and dropconnect [14,15]. Gal and Ghahramani [16] could show that an appropriate application of Bernoulli dropout can be interpreted as training networks in a Bayesian way, by approximating the a posteriori distribution via variational inference. Kingma et al. [17] showed the same also for Gaussian dropout.

Both major drawbacks of standard deep learning, the absence of model uncertainty information and the need of a large amount of training data, are well addressed by using Bayesian statistics. On the one hand Bayesian models are robust to overfitting since parameters are not forced to be fixed and on the other hand the uncertainty in the network parameters can directly be translated in uncertainty information for network predictions. Further, Bayesian deep models can help to finally understand why deep learning works. Combining the profound theoretical literature about Bayesian statistics and deep learning will lead to a better understanding and broader acceptance of the technique. Whereas nowadays, most network architectures are designed based on trial and error or based on abstract, high level considerations [18,19], the insights gained from model uncertainty information may enable designing optimal architectures in an analytical fashion. For a neat account on Deep Learning frameworks from a Bayesian perspective we refer to Polson and Sokolov [20]. It should be pointed out that Bayesian methods to quantify uncertainties for the parameters of neural networks and their predictions [21,22,23] predate the advent of deep learning. In Reference [21], a Markov chain Monte-Carlo sampling strategy is adopted that samples from a network architecture dependent potential energy function approximating the posterior distribution of the network parameters. MacKay [22], amongst other things, introduces the concept of automatic relevance determination priors to control inputs to the neural network based on their support in the training data. v. Toussaint et al. [23] derive a hyperplane prior for the network weights that satisfies the required transformation invariance. They calculate the intractable evidence integral in their Bayesian framework using a second order Laplace approximation. Finally, we would also like to mention that there are approaches based on classical deep learning to determine the uncertainty of the predictions. In particular Lakshminarayanan et al. [24] show that accurate uncertainty information can be obtained by evaluating the predictions of ensembles of classical networks. This comes at the expense of training and evaluating multiple models for the same task.

In this study, a new approach for Bayesian deep learning based on variational inference is proposed. In particular, the proposed technique treats network layers as units in order to express model uncertainty. Therefore, only two uncertainty parameters are introduced per layer which implies that the variational distribution requires only few additional parameters that need to be optimized compared to a non-Bayesian net. Thus, while allowing for an easy, intuitive layer-wise analysis of model uncertainty, the network optimization should not become significantly harder. According to Gal and Ghahramani [12] the introduction of many additional parameters results in architectures that require more time to converge and that do not improve on existing approaches.

## 2. Variational Inference and Related Work

In this section, a short introduction to Bayesian statistics and variational inference is presented. More details can be found in References [12,25,26,27,28,29]. Further, how variational inference was applied in the past to train deep neural networks in a Bayesian way is summarized, which also sheds some light on the limitations of each approach.

### 2.1. Bayesian and Variational Inference

The theoretical considerations are based on classification tasks in this study. For regression, the theory is quite the same and can be found in the literature recommended above. In Bayesian statistics, network parameters are considered as one large random vector W. A priori knowledge regarding W is expressed in terms of the a priori distribution p(w). One is interested in updating the knowledge about W after observing data D={y,X}, where $X=\{x_{1},...,x_{\beta }\}$ denotes a set of training examples and $y={\left(y_{1},...,y_{\beta }\right)}^{T}$ holds the corresponding class labels. Therefore, the a posteriori distribution p(w|y,X) has to be calculated. According to the Bayes’ theorem, the corresponding density is

(1) $$\begin{array}{c} p\left(w|y,X\right)=\frac{p(y|w,X)p(w)}{\int p(y|w,X)p(w)\;dw}. \end{array}$$

The probability p(y|w,X) is given by the product of the neural network outputs for all the training examples, following the classical assumptions on stochastic independence and modeling in deep learning for classification tasks. Thus, the only problem in computing p(w|y,X) is the generally intractable integral in the denominator. Variational inference aims at approximating the posterior p(w|y,X) by optimizing a parametric distribution $q_{\phi }\left(w\right)$, such that it is most similar to p(w|y,X).

Once the variational distribution is optimized, it can be used for the prediction of new data and, further, for quantifying uncertainty about predictions. The posterior predictive distribution $p(y^{*}|x^{*},y,X)$ reflects the belief in a class label $y^{*}$ for a given example $x^{*}$ after observing data y,X:

(2) $$\begin{array}{c} \begin{array}{cc} p(y^{*}|x^{*},y,X) & =\int p(y^{*},w|x^{*},y,X)\;dw\\ \; & =\int p\left(y^{*}|w,x^{*},y,X\right)p\left(w|y,X\right)\;dw\\ \; & =\int p\left(y^{*}|w,x^{*}\right)p\left(w|y,X\right)\;dw. \end{array} \end{array}$$

Replacing the posterior with the variational distribution and further approximating the intractable integral via Monte Carlo integration results in

(3) $$\begin{array}{c} p\left(y^{*}|x^{*},y,X\right)\approx \frac{1}{N}\sum_{i=1}^{N}f{\left(x^{*};w_{i}\right)}_{y^{*}},\;\;\;\mathrm{with}\;w_{i}\underset{i.i.d.}{∼}q_{\phi }\left(w\right), \end{array}$$

where f denotes the neural network used. Thus, predictions $\hat{y^{*}}$ are made by propagating the object of interest $x^{*}$ multiple times through the network, averaging the resulting probability vectors and choosing the index of the largest element in the resulting mean:

(4) $$\begin{array}{c} \hat{y^{*}}=\underset{j}{arg max}\frac{1}{N}\sum_{i=1}^{N}f{\left(x^{*};w_{i}\right)}_{j}. \end{array}$$

The posterior predictive distribution incorporates model uncertainty by averaging over the variational distribution. Besides investigating $p(y^{*}|x^{*},y,X)$, in order to quantify prediction uncertainty, one can also estimate credible intervals for the probability that a given instance $x^{*}$ corresponds to a given class $y^{*}$. To this aim, one has to sample from the variational distribution and subsequently calculate the empirical $\frac{\alpha }{2}$ and $1-\frac{\alpha }{2}$ quantiles of the corresponding network outputs. As a result, one obtains an estimate of the 1−α credible interval for the probability that $x^{*}$ belongs to class $y^{*}$ is found. Note that the a posteriori uncertainty about the random network parameters W induces uncertainty about the neural network output $f{\left(x^{*},W\right)}_{y^{*}}$, that is, the probability of an object $x^{*}$ belonging to class $y^{*}$.

So far it has not been mentioned how the variational distribution is optimized in order to approximate the posterior p(w|y,X). This can be accomplished by minimizing the Kullback-Leibler divergence (*KL-divergence*) $D_{KL}(q_{\phi }\left(w\right)||p\left(w|y,X\right))$ between the variational distribution and the posterior. It is defined as:

(5) $$\begin{array}{c} \begin{array}{cc} D_{KL}(q_{\phi }\left(w\right)||p\left(w|y,X\right)) & =E_{q_{\phi }\left(w\right)}\left(\ln\frac{q_{\phi }\left(w\right)}{p(w|y,X)}\right)\\ \; & =\int \ln\frac{q_{\phi }\left(w\right)}{p(w|y,X)}q_{\phi }\left(w\right)\;dw. \end{array} \end{array}$$

The KL-divergence is not really a distance measure since it is asymmetric and the triangle inequality does not hold. Nevertheless, it is often used to measure the distance between two probability distributions, and as long as only two distributions are of interest it does not matter that the triangle inequality is violated. Obviously, $D_{KL}(q_{\phi }\left(w\right)||p\left(w|y,X\right))$ cannot be minimized directly since the a posteriori distribution is unknown. However, minimizing $D_{KL}(q_{\phi }\left(w\right)||p\left(w|y,X\right))$ is equivalent to minimizing the negative log evidence lower bound $L_{VI}$ [28], which is given by:

(6) $$\begin{array}{c} \begin{array}{cc} L_{VI} & =-\int q_{\phi }\left(w\right)\ln p\left(y|w,X\right)\;dw+D_{KL}(q_{\phi }\left(w\right)||p\left(w\right))\\ \; & =-E_{q_{\phi }\left(w\right)}\left[\ln\prod_{i=1}^{\beta }p\left(y_{i}|w,x_{i}\right)\right]+D_{KL}(q_{\phi }\left(w\right)||p\left(w\right))\\ \; & =-\sum_{i=1}^{\beta }\left[E_{q_{\phi (w)}}\left(\ln p(y_{i}|w,x_{i})\right)\right]+D_{KL}(q_{\phi }\left(w\right)||p\left(w\right)). \end{array} \end{array}$$

The lower bound $L_{VI}$ includes the KL-divergence between the variational distribution and the well known prior. The unknown expectation value can be approximated via Monte Carlo integration. Inspired by stochastic gradient descent the integration takes place with just one sample, but a new sample is drawn in each iteration of the optimization procedure used to minimize $L_{VI}$. The re-sampling guarantees that a sufficient amount of samples is drawn, whilst using merely one sample saves memory. According to these considerations, the objective function in the *k*-th iteration of the optimization is given by:

(7) $$\begin{array}{c} {\hat{L}}_{VI}=-\sum_{i=1}^{\beta }\{\ln f{\left(x_{i};w_{k}\right)}_{y_{i}}\}+D_{KL}(q_{\phi }\left(w\right)||p\left(w\right)). \end{array}$$

If one wants to use mini-batch gradient descent, the KL-divergence has to be re-scaled by the factor $\frac{m}{\beta }$, where *m* denotes the number of examples one mini-batch holds. This ensures that the divergence does not get too much weight.

Summing up, training neural networks in a Bayesian way via variational inference is quite similar to frequentist training. The L2-norm regularization used in classical deep learning is replaced by punishing deviations from the a priori distribution. The same loss function as in non-Bayesian deep learning is applied, but with the crucial difference that the network parameters are drawn from the variational distribution during training.

### 2.2. Related Work

There is a large number of possibilities to define the variational distribution. Gal and Ghahramani [16] have shown that classical Bernoulli dropout can be used to define the approximating function. The network biases are assumed to be deterministic for simplicity, whilst the network weights are defined to be random according to dropout. Indeed, randomly dropping a neuron in layer i−1 is equivalent to dropping all weights in layer *i* which represent connections to this one neuron. In order to calculate the KL-divergence to a standard normal prior, network weights are assumed to follow a mixture of two Gaussians. Note that the KL-divergence between a discrete and a continuous distribution would diverge to infinity. Both Gaussians are defined to have a variance that is negligibly small, such that more or less only two values ( zero and a variational parameter to be optimized ) are taken. Finally, the KL-divergence is given by the L2-norm of the neural network weights. Therefore, neural nets can be learned in a Bayesian way by merely applying Bernoulli dropout before each weight layer. Experiments have shown that this approach results in a very good accuracy at the MNIST dataset of handwritten digits [16]. While Gal and Ghahramani [16] considered Bernoulli dropout, Kingma et al. [17] investigated Gaussian dropout in terms of Bayesian deep learning. Instead of dropping neurons in layer i−1 in this approach the output vector $a=(a_{1},...,a_{R})$ of layer i−1 is component-wise multiplied with a vector $\xi =(\xi_{1},...,\xi_{R})$, where the ξ are i.i.d. distributed according to $N(1,\tau^{2})$. Let $M\in R^{R\times S}$ denote the weight matrix of layer *i* (assumed to be a fully-connected layer), that is, the matrix that maps the output vector $a=(a_{1},...,a_{R})$ of layer i−1 to the *S* neurons of layer *i*. Further, let ⊙ denote the Hadamard product. Then the product a⊙ξM can be written as

(8) $$\begin{array}{c} \left(a_{1},...,a_{R}\right)\left(\begin{array}{ccc} \underset{=W_{11}}{\underbrace{m_{11}\xi_{1}}} & \cdots & \underset{=W_{1S}}{\underbrace{m_{1S}\xi_{1}}}\\ \; & \vdots & \\ \underset{=W_{R1}}{\underbrace{m_{R1}\xi_{R}}} & \cdots & \underset{=W_{RS}}{\underbrace{m_{RS}\xi_{R}}} \end{array}\right). \end{array}$$

Consequently, using Gaussian dropout can be interpreted as approximate Bayesian inference with a Gaussian variational distribution assigned to the weights. The marginal distribution of a given weight $W_{rs}$ is given by $N(m_{rs},\tau^{2}m_{rs}^{2})$, weights corresponding to different neurons of layer i−1 are independent, and weights that point to the same neuron of layer i−1 have correlation

(9) $$\begin{array}{c} \begin{array}{cc} \mathrm{Cor}(W_{rs},W_{rk}) & =\frac{\mathrm{Cov}(m_{rs}\xi_{r},m_{rk}\xi_{r})}{\sqrt{\mathrm{Var}(m_{rs}\xi_{r})}\sqrt{\mathrm{Var}(m_{rk}\xi_{r})}}\\ \; & =\frac{m_{rs}m_{rk}\mathrm{Var}\left(\xi_{r}\right)}{|m_{rs}m_{rk}|\mathrm{Var}\left(\xi_{r}\right)}\\ \; & =\mathrm{sign}(m_{rs}m_{rk}). \end{array} \end{array}$$

Blundell et al. [30] used a normal distribution with a diagonal covariance matrix as variational distribution. The approach was evaluated with the LeNet architecture [31] and the MNIST dataset. While the approach was shown to work in principle, a wider application is hampered by the fact that the number of parameters to be optimized is doubled *(one variance term for each expectation value)* which complicates training and makes it computationally significantly more expensive, as stated by Gal and Ghahramani [12].

Louizos and Welling [32] introduced a variational distribution that in contrast to the distribution of Blundell et al. does not treat each network parameter independently. In particular, they used a probability distribution on random matrices. Thus, they could reduce the number of variance-related parameters, but to a number which nonetheless is significantly higher than in the frequentist approach. Another approach with similar restrictions is described in Reference [17].

It should be mentioned that variational Bayes is just a specific case of local α-divergence minimization. The α-divergence [33] between two densities p(w) and q(w) is defined as

(10) $$\begin{array}{c} D_{\alpha }\left(p\left(w\right)||q\left(w\right)\right)=\frac{1}{\alpha (1-\alpha )}\left(1-\int p{\left(w\right)}^{\alpha }q{\left(w\right)}^{1-\alpha }\;dw\right), \end{array}$$

such that $D_{\alpha }\left(p\left(w\right)||q\left(w\right)\right)$ converges to the Kullback-Leibler divergence $D_{KL}\left(q\left(w\right)||p\left(w\right)\right)$ for α→0. Hernandez-Lobato et al. [34] have shown that the optimal setting for α is task specific and that a nonstandard stetting α≠0 can produce better prediction results. Li and Gal [35] continued the work of Reference [34]. According to them, variational inference can underestimate model uncertainty and α-divergences are able to avoid the underestimation. In particular they propose a simple inference technique based on a reparameterization of the α-divergence objectives and dropout. However, our work does not focus on finding an optimal choice for α. It tries to propose a good and reasonable approximating distribution. The proposed distribution can then be used with any setting of α, but this is left for further research. In a recent study published by some of the authors of this manuscript [36], the variational distribution is modeled as multiple multivariate normal distributions with tridiagonal covariance matrices. While this approach allows for network parameters to be correlated, it also adds to the computational complexity compared to the approach presented here that assumes diagonal covariance matrices.

## 3. Materials and Methods

In this work, we propose a variational distribution with the aim to satisfy the following two requirements:

- useful prediction uncertainty information can be computed
- the number of parameters to be optimized does not differ significantly from the non-Bayesian case

In order to satisfy these requirements, our approach expresses model uncertainty layer-wise with respect to the parameter expectation values. Therefore, only two uncertainty parameters are introduced per layer. Consequently, the number of parameters to optimize compared to a standard network is quite small, which is a desirable property according to Gal and Ghahramani [12]. When studying Section 3.1 the reader will notice that our variational distribution is quite similar to the one induced by Gaussian Dropout [17]. However, there are two crucial differences. Gaussian dropout implies that weights that point to the same neuron are correlated with correlation 1 if their expected values have the same sign and with correlation −1 otherwise. Since allowing for learnable correlations in the variational distribution enables a better approximation of the true posterior the consideration of such dependence structures is generally useful. Nevertheless, we are unsure if the correlations implied by Gaussian Dropout are helpful, since they are fixed and maximal, that is, their absolute value is equal to one. The variational distribution proposed by us assumes complete independence between all network parameters. The second second main difference to the work of Kingma et al. [17] is that in our approach also the bias terms are interpreted as random variables.

### 3.1. Derivation of the Approach

Let $W_{i}={\left(W_{i1},...,W_{iK_{i}}\right)}^{T}$ denote the random weights of the *i*-th network layer and, further, let $B_{i}=\left(B_{i1},...,B_{ik_{i}}\right)$ denote the corresponding random biases. In addition, let $\varepsilon_{i}={\left(\varepsilon_{i1},...,\varepsilon_{iK_{i}}\right)}^{T}$ and $\varepsilon_{bi}={\left(\varepsilon_{bi1},...,\varepsilon_{bik_{i}}\right)}^{T}$ be multivariate standard normal distributed, where the subscript *b* is used to identify expressions related to the biases of the network. To set up the variational distribution, the random weights $W_{i}$ and the random biases $B_{i}$ are defined by

(11) $$\begin{array}{cc} W_{i} & =m_{i}⊙\left(1_{K_{i}}+\tau_{i}\varepsilon_{i}\right), \end{array}$$

(12) $$\begin{array}{cc} \tau_{i} & =\ln(1+\exp\left(\delta_{i}\right)), \end{array}$$

(13) $$\begin{array}{cc} B_{i} & =m_{bi}⊙\left(1_{k_{i}}+\tau_{bi}\varepsilon_{bi}\right), \end{array}$$

(14) $$\begin{array}{cc} \tau_{bi} & =\ln(1+\exp\left(\delta_{bi}\right)), \end{array}$$

where $m_{i}\in R^{K_{i}}$, $m_{bi}\in R^{k_{i}}$, $\delta_{i}\in R$ and $\delta_{bi}\in R$ are variational parameters and ⊙ denotes the Hadamard product, that is, element-wise multiplication. This implies that $W_{i}$ and $B_{i}$ are multivariate normal distributed according to

(15) $$\begin{array}{cc} W_{i} & ∼N\left(m_{i},\tau_{i}^{2}\mathrm{diag}{\left(m_{i}\right)}^{2}\right), \end{array}$$

(16) $$\begin{array}{cc} B_{i} & ∼N(m_{bi},\tau_{bi}^{2}\mathrm{diag}{\left(m_{bi}\right)}^{2}). \end{array}$$

The reason why weights and biases are not directly defined by the Gaussians given in Equations (15) and (16), can be found in the network optimization. During optimization in each iteration, a sample is drawn from the random network parameters in order to adjust the variational parameters by gradient descent. A direct sampling from Equations (15) and (16) would mask the variational parameters and therefore exclude them from optimization. The indirect sampling according to Equations (11) and (13) ensures that the variational parameters can be updated. Generally, the idea of expressing the random network parameters by means of a differentiable function of an (auxiliary) noise variable is known as the reparameterization trick [37]. In addition, $\tau_{i}$ and $\tau_{bi}$ are reparameterized with help of the softplus function (Equations (12) and (14)). These parameters regulate the variances of the variational distribution and, therefore, should not take negative values. Finally we define the overall variational distribution by

(17) $$\begin{array}{c} \begin{array}{cc} q_{\phi }\left(w\right) & =\prod_{i=1}^{d}q_{\phi_{i}}\left(w_{i}\right)q_{\phi_{bi}}\left(b_{i}\right),\\ \mathrm{with}\;\phi_{i} & =\left\{m_{i},\delta_{i}\right\},\;\mathrm{and}\;\phi_{bi}=\left\{m_{bi},\delta_{bi}\right\}, \end{array} \end{array}$$

by assuming that $W_{1},\ldots ,W_{d}$ and $B_{1},\ldots ,B_{d}$ are statistically independent and with $q_{\phi_{i}}\left(w_{i}\right)$ and $q_{\phi_{bi}}\left(b_{i}\right)$ denoting densities of normal distributions according to Equations (15) and (16). The depth of the network is denoted by *d*. Thus, parameter uncertainty is expressed layer-wise and relative to the parameter expectation values. In analogy to the variational density, the normal prior p(w) is defined as

(18) $$\begin{array}{cc} p(w) & =\prod_{i=1}^{d}p\left(w_{i}\right)p\left(b_{i}\right), \end{array}$$

with $W_{i}∼N\left(\mu_{i},\zeta_{i}^{2}I_{K_{i}\times K_{i}}\right)$ and $b_{i}∼N\left(\mu_{bi},\zeta_{bi}^{2}I_{k_{i}\times k_{i}}\right)$. Therefore, the a priori uncertainty is again expressed layer-wise. The fact that both the variational distribution and the prior factorize simplifies the calculation of the Kullback-Leibler divergence between those two. It is given by the sum of the layer-wise divergences:

(19) $$\begin{array}{c} \begin{array}{cc} D_{KL}(q_{\phi }\left(w\right)||p\left(w\right)) & =E_{q_{\phi }\left(w\right)}\left(\ln\frac{\prod_{i=1}^{d}q_{\phi_{i}}\left(w_{i}\right)q_{\phi_{bi}}\left(b_{i}\right)}{\prod_{i=1}^{d}p\left(w_{i}\right)p\left(b_{i}\right)}\right)\\ \; & =E_{q_{\phi }\left(w\right)}\left\{\sum_{i=1}^{d}\left[\ln\left(q_{\phi_{i}}\left(w_{i}\right)\right)-\ln\left(p\left(w_{i}\right)\right)\right]\right.\\ \; & \left.+\sum_{i=1}^{d}\left[\ln\left(q_{\phi_{bi}}\left(b_{i}\right)\right)-\ln\left(p\left(b_{i}\right)\right)\right]\right\}\\ \; & =\sum_{i=1}^{d}\left[E_{q_{\phi_{i}}\left(w_{i}\right)}\ln\frac{q_{\phi_{i}}\left(w_{i}\right)}{p(w_{i})}\right]+\sum_{i=1}^{d}\left[E_{q_{\phi_{bi}}\left(b_{i}\right)}\ln\frac{q_{\phi_{bi}}\left(b_{i}\right)}{p(b_{i})}\right]\\ \; & =\sum_{i=1}^{d}\left[D_{KL}(q_{\phi_{i}}\left(w_{i}\right)||p\left(w_{i}\right))\right]+\sum_{i=1}^{d}\left[D_{KL}(q_{\phi_{bi}}\left(b_{i}\right)||p\left(b_{i}\right))\right]. \end{array} \end{array}$$

Given that the KL-divergence between two *p*-dimensional Gaussians $h\left(x\right)=N\left(x;\mu_{h},\Sigma_{h}\right)$ and $g\left(x\right)=N\left(x;\mu_{g},\Sigma_{g}\right)$ is given by [38]

(20) $$\begin{array}{c} \begin{array}{cc} D_{KL}\left(h||g\right)=\frac{1}{2} & \left[\ln\frac{|\Sigma_{g}|}{|\Sigma_{h}|}+\mathrm{tr}\left(\Sigma_{g}^{-1}\Sigma_{h}\right)-p\right.\\ \; & \left.+{\left(\mu_{h}-\mu_{g}\right)}^{T}\Sigma_{g}^{-1}\left(\mu_{h}-\mu_{g}\right)\right], \end{array} \end{array}$$

where |X| means the determinant of X, it is easy to calculate $D_{KL}(q_{\phi }\left(w\right)||p\left(w\right))$. It is given by:

(21) $$\begin{array}{c} \begin{array}{cc} D_{KL}(q_{\phi }\left(w\right) & ||p(w))=\\ \; & \frac{1}{2}\sum_{i=1}^{d}\left[-\ln(\tau_{i}^{2K_{i}}|\mathrm{diag}{\left(m_{i}\right)}^{2}\left|\right)+\frac{\tau_{i}^{2}}{\zeta_{i}^{2}}\left|\right|m_{i}{\left|\right|}_{2}^{2}+\frac{1}{\zeta_{i}^{2}}\left|\right|m_{i}-\mu_{i}{\left|\right|}_{2}^{2}\right.\\ \; & \left.-\ln(\tau_{bi}^{2k_{i}}|\mathrm{diag}{\left(m_{bi}\right)}^{2}\left|\right)+\frac{\tau_{bi}^{2}}{\zeta_{bi}^{2}}\left|\right|m_{bi}{\left|\right|}_{2}^{2}+\frac{1}{\zeta_{bi}^{2}}\left|\right|m_{bi}-\mu_{bi}{\left|\right|}_{2}^{2}\right]\\ \; & +\frac{1}{2}\sum_{i=1}^{d}\left[\ln\left(\zeta_{i}^{2K_{i}}\right)+\ln\left(\zeta_{bi}^{2k_{i}}\right)-K_{i}-k_{i}\right], \end{array} \end{array}$$

where the last sum is an additive constant that plays no role for the optimization.

In order to train a network f according to our approach the partial derivatives of f with respect to $m_{ij},m_{bij},\delta_{i}$ and $\delta_{bi}$ are needed. These derivatives can easily be expressed in terms of the classical derivatives in non-Bayesian deep learning. According to the chain rule the derivatives with respect to $m_{ij}$ and $m_{bij}$ are given by

(22) $$\begin{array}{cc} \frac{\partial f}{\partial m_{ij}} & =\frac{\partial f}{\partial w_{ij}}\cdot \frac{\partial w_{ij}}{\partial m_{ij}}=\frac{\partial f}{\partial w_{ij}}\cdot \left(1+\tau_{i}\varepsilon_{ij}\right), \end{array}$$

(23) $$\begin{array}{cc} \frac{\partial f}{\partial m_{bij}} & =\frac{\partial f}{\partial b_{ij}}\cdot \frac{\partial b_{ij}}{\partial m_{bij}}=\frac{\partial f}{\partial b_{ij}}\cdot \left(1+\tau_{bi}\varepsilon_{bij}\right), \end{array}$$

where $\frac{\partial f}{\partial w_{ij}}$ and $\frac{\partial f}{\partial b_{ij}}$ are calculated as in the non-Bayesian case. Note that

(24) $$\begin{array}{cc} w_{ij} & =m_{ij}\left(1+\tau_{i}\varepsilon_{ij}\right), \end{array}$$

(25) $$\begin{array}{cc} b_{ij} & =m_{bij}\left(1+\tau_{bi}\varepsilon_{bij}\right), \end{array}$$

are denoting samples from $W_{ij}$ and $B_{ij}$, respectively. Analogously, the derivatives with respect to $\delta_{i}$ and $\delta_{bi}$ are given by:

(26) $$\begin{array}{cc} \frac{\partial f}{\partial \delta_{i}} & =\sum_{j}\frac{\partial f}{\partial w_{ij}}\cdot m_{ij}\varepsilon_{ij}\frac{e^{\delta_{i}}}{1+e^{\delta_{i}}}, \end{array}$$

(27) $$\begin{array}{cc} \frac{\partial f}{\partial \delta_{bi}} & =\sum_{j}\frac{\partial f}{\partial b_{ij}}\cdot m_{bij}\varepsilon_{bij}\frac{e^{\delta_{bi}}}{1+e^{\delta_{bi}}}. \end{array}$$

Further, one can easily verify that the partial derivatives of the KL-divergence $D_{KL}$ are given by:

(28) $$\begin{array}{cc} \frac{\partial }{\partial m_{ij}}D_{KL} & =-\frac{1}{m_{ij}}+\frac{\tau_{i}^{2}}{\zeta_{i}^{2}}m_{ij}+\frac{1}{\zeta_{i}^{2}}\left(m_{ij}-\mu_{ij}\right), \end{array}$$

(29) $$\begin{array}{cc} \frac{\partial }{\partial \delta_{i}}D_{KL} & =\frac{\exp(\delta_{i})}{1+\exp(\delta_{i})}\left[\frac{\tau_{i}}{\zeta_{i}}\left|\right|m_{i}{\left|\right|}_{2}^{2}-\frac{K_{i}}{\tau_{i}}\right], \end{array}$$

(30) $$\begin{array}{cc} \frac{\partial }{\partial m_{bij}}D_{KL} & =-\frac{1}{m_{bij}}+\frac{\tau_{bi}^{2}}{\zeta_{bi}^{2}}m_{bij}+\frac{1}{\zeta_{bi}^{2}}\left(m_{bij}-\mu_{bij}\right), \end{array}$$

(31) $$\begin{array}{cc} \frac{\partial }{\partial \delta_{bi}}D_{KL} & =\frac{\exp(\delta_{bi})}{1+\exp(\delta_{bi})}\left[\frac{\tau_{bi}}{\zeta_{bi}}\left|\right|m_{bi}{\left|\right|}_{2}^{2}-\frac{k_{i}}{\tau_{bi}}\right]. \end{array}$$

### 3.2. Implementation

We implemented the approach illustrated above in two ways: (i) by modifying the popular open-source Caffe framework [39,40] and (ii) by writing custom layer definitions for the pytorch framework [41]. In the former, the layer parameter “blobs” of the convolutional layer and the inner product layer were extended to include the additional variance terms for the weights and biases, $\delta_{i}$ and $\delta_{bi}$ respectively, as well as the current realizations of $\varepsilon_{i}$ and $\varepsilon_{bi}$. The weights and biases of the classical implementation are here interpreted as the variational parameters $m_{i}$ and $m_{bi}$. In addition, for each layer, static arrays to hold the prior expectation values for the weights and biases $\mu_{ij}$ and $\mu_{bij}$ and the corresponding a priori variance terms $\zeta_{i}$ and $\zeta_{bi}$ were introduced. The variances and expectation values of the prior distributions and the starting values for the variances and expectation values of the variational distributions $\delta_{i}$ and $\delta_{bi}$ can be set for each layer in the network definition prototext file. During each forward pass, one sample is drawn from the $\varepsilon_{i}$ and the $\varepsilon_{bi}$. From this, the random weights and biases used in the forward pass, $W_{i}$ and $B_{i}$, are calculated according to Equations (11)–(14) using the current variational parameters and the current realizations of $\varepsilon_{i}$ and $\varepsilon_{bi}$. During the backward pass, the gradients of the weights and biases are adapted according to Equations (22) and (23). In addition, the gradients for the new variance parameters are calculated according to Equations (26) and (27). For each gradient, the additional term due to the Kullback-Leibler divergence is added following Equations (28)–(31). These gradients are then used to calculate the updated variational parameters to be used for the next forward pass. The implementation for the pytorch framework is more straightforward: A custom python module holds class definitions for the 2D convolutional and the inner product layer. Both inherit the basic functionality from the corresponding pytorch classes. In the class initialization routines, the additional parameters are registered as model parameters and the arrays of random numbers are drawn from a zero-mean, unit variance normal distribution. The forward function of each class is modified to draw the new random weights and biases which are then passed to the forward function of the superclass. In the backward pass, the autograd engine of pytorch takes care of computing the partial derivatives for the additional model parameters. The regularization term due to the Kullback-Leibler divergence is added to the gradient of each additional parameter with the help of backhook functions that are executed once the basic gradient has been computed by the autograd engine. To avoid large correction terms caused by small values, the regularization term of the Kullback-Leibler divergence is limited to the interval of −1000 to 1000. In terms of the resulting computational complexity of our proposed approach, we note that the number of parameters to be optimized is essentially the same as only two parameters per layer are added. For example, for the LeNet architecture used in this study, the frequentist formulation already contains more than 430,000 parameters for optimization while our Bayesian approach only adds 8 additional parameters. The number of required MAC operations differ significantly between the two approaches. This is mostly due to the random sampling of the network parameters in each forward pass. The number of additional MAC operations required can be estimated from Equations (11)–(14). Thus, we can expect the number of MAC operations for our suggested approach to be roughly 6-times higher compared to the frequentist approach.

## 4. Results and Discussion

In this section, the performance of our approach is evaluated in different ways and compared to other approaches. In Section 4.1, we illustrate the performance using a simple architecture (LeNet [31]) and dataset (MNIST [42]). We compare the results we obtain with classical deep learning based on frequentist interpretation and the approach proposed by Gal and Ghahramani [16] that uses Bernoulli dropout to define the variational distribution. Since we expect Bernoulli dropout to perform similar to Gaussian dropout [17] due to the Central Limit Theorem, we do not include a comparison against the latter approach. We also analyze the uncertainty information obtained about the network parameters as well as the network predictions in detail. These results were obtained using the caffe implementation described above. In Section 4.2, we compare our approach to the “Bayes by Backprop” algorithm [30] and show that we can achieve comparable or better results despite giving up some flexibility in representing the true posterior uncertainty of the network parameters. Finally, in Section 4.3, we illustrate the benefits of trading off flexibility for a lower number of parameters by showing that our approach can also be used for larger network architectures with larger input data vectors. This is illustrated by fine-tuning a GoogLeNet [43] architecture with a custom dataset. The last two results were obtained with the pytorch implementation.

### 4.1. LeNet and the MNIST Dataset

Basis of the experiments in this section is the benchmark dataset MNIST [42] together with the architecture LeNet [31]. The MNIST dataset consists of 70,000 images of handwritten digits, from which 60,000 build up the training dataset and the remaining 10,000 build up the testing data. The specific version of LeNet used is the same described in Reference [16]. Therefore the first convolutional layer generates 20 feature maps, while the second one extracts 50 features. Both layers use (5×5) kernels. Max-pooling with kernel size (2×2) and stride 2 is applied after both convolutional layers. The first fully connected layer consists of 500 neurons, the second one covers only 10 since there are 10 different digits. Moreover, the first fully connected layer uses the rectified linear unit as activation and the other ones the identity function.

In order to get an idea how well our Bayesian approach performs it should be compared to the classical, that is, the frequentist, approach. Therefore, LeNet is trained three times in the classical way. First, without dropout, then with dropout ( dropping rate 0.5) applied after the first inner product layer, and finally, with dropout applied as before and exchanged training and testing datasets. Exchanging training and testing data results in a significant reduction of the training data from 60,000 to 10,000 and should give an intuition how well Bayesian models work for limited training data.

All three models are optimized the same way. To prevent overfitting, the Euclidean norm of the network weights is penalized with a factor of 0.0005. As usual in deep learning, the optimization procedure applied is mini-batch gradient descent. A batch size of 64 is chosen. The learning rate used in the *i*-th iteration is given by $0.01*{\left(1+0.0001*i\right)}^{-0.75}$. Momentum is used and set to 0.9. The accuracies for the test dataset are given in Table 1 and are expressed by the corresponding test error defined as $1-\frac{N_{corr}}{N}=1-accuracy$ where $N_{corr}$ is the number of correctly classified samples and *N* is the total number of samples in the test dataset (here N= 10,000).

**Figure 1:**
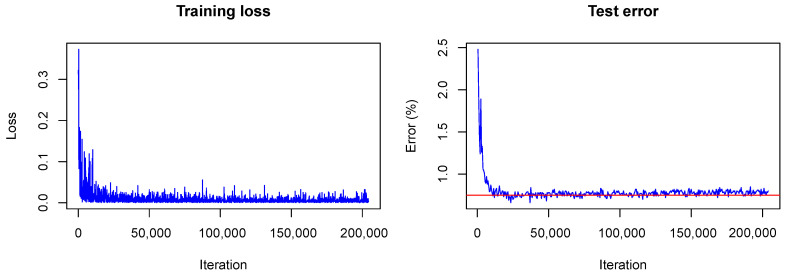
Training visualization of frequentist LeNet with dropout. The horizontal line marks the achieved accuracy.

**Figure 2:**
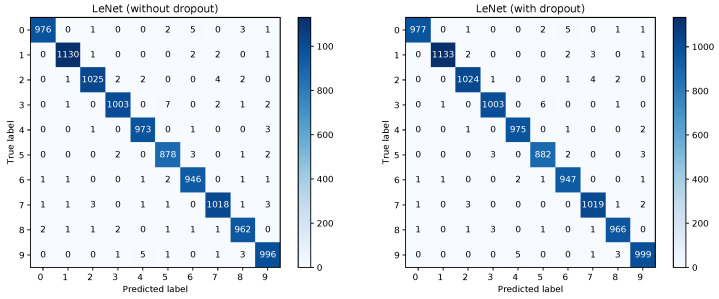
Confusion matrices for the frequentist LeNet without and with dropout.

**Figure 3:**
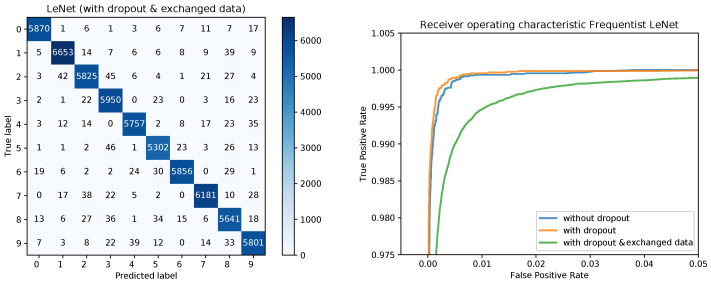
Confusion matrix for the frequentist LeNet with dropout and exchanged data and ROC curves for all three models.

The training converged quite similarly in all three cases. A visualization of the training loss and test error for the second model, that is, the model trained with dropout, is shown in   Figure 1. This figure will serve for comparison of the Bayesian and the frequentist training process. A plot of the confusion matrices is shown in Figure 2 for the models without dropout (left) and with dropout (right) and in Figure 3 (left) for the the model with dropout and exchanged training and testing data. The right side of Figure 3 shows a zoomed in section of the ROC curves for the three models. Each ROC curve is the mean of the one-against-all ROC curves for each class. The results confirm the performance differences of the different models as indicated by Table 1.

Similarly for our Bayesian approach, LeNet is trained three times with the MNIST dataset. In analogy to the frequentist training (see above), LeNet is trained first without dropout, then with dropout, and finally, with dropout and exchanged training and testing data. In contrast to Gal and Ghahramani, we interpret dropout training as simultaneous training of multiple Bayesian models and assume that combining multiple models will result in a better accuracy than using just one model. Thus, during testing, the weight scaling inference rule, which states that each neuron should be used but multiplied with the dropping ratio, is not applied. Rather in the testing phase, neurons are randomly dropped in order to sample from the set of simultaneously trained Bayesian models and combine their predictions to one overall prediction.

**Figure 4:**
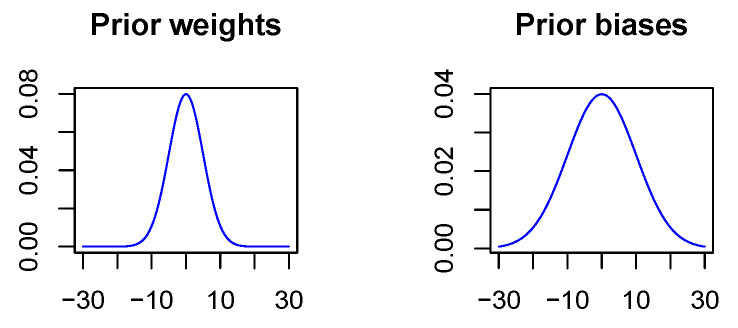
Visualization of the a priori distribution. Bias terms may take on larger values since they act on sums.

In contrast to the non-Bayesian case, a penalization of the Euclidean norm does not take place since in the Bayesian case deviations from the a priori distribution are penalized. As there is not really a priori information available, the prior is used to express the wish that values should not diverge. Thus, the a priori expectation value is specified as zero for all network parameters, and further, the a priori standard deviation is chosen to be 5 for all weights and 10 for all biases. The variance for the biases is chosen to be larger since biases act on linear combinations of neuron outputs with network weights as coefficients and therefore may take on larger values, see  Figure 4. It should be mentioned that the penalization strength of the KL-divergence between the variational distribution and the a priori distribution is chosen smaller than recommended in the theoretical considerations in Section 3.1 because of convergence problems. Empirically, we found that we have to scale the penalization strength down by a factor of 100 to ensure convergence. While somewhat puzzling, this does not matter since there is not really a priori information available and the network parameters took small values in all experiments even with the reduced penalization. It should also be mentioned, that our implementation easily lends itself to ’Bayesian transfer learning’ in analogy to classical transfer learning [44], where the results of a previous training run with a large dataset are optimized for a more targeted application by fine-tuning the network with a smaller but specific training dataset. In the Bayesian case, the information about the posterior distribution of the network parameters in the pre-trained network will then be used to specify a prior for the fine-tuning step. This is subject to future work.

**Figure 5:**
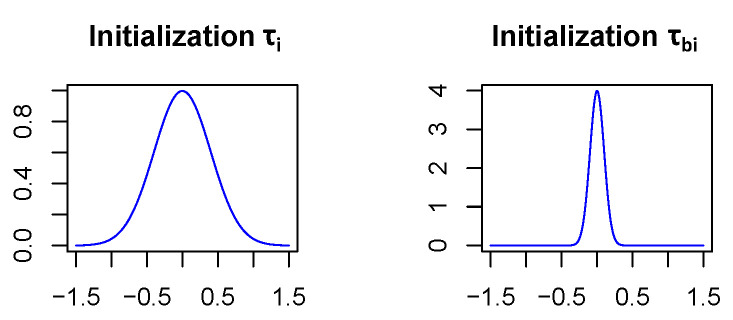
Visualization of the initialization of $\tau_{i}$ and $\tau_{bi}$. Network weights can differ at most by the size of their expectation value from their expectation value.

In order for the Bayesian networks to converge, the parameters $\tau_{i}$ and $\tau_{bi}$ (see Section 3.1) which specify the standard deviations of the network weight and bias distributions, respectively, have to be initialized carefully. Therefore, $\tau_{i}$ is initialized as 0.4 and $\tau_{bi}$ as 0.1 in all network layers except for the first fully connected one which is treated separately. In this initialization, it becomes highly unlikely (<0.7%) that the weights of the neural net differ by more than the size of their expectation value from their expectation value, see  Figure 5. This is a reasonable way to start since stronger deviations from the expectation values would mean that weights are even unsure about their algebraic sign, which might lead to convergence issues, if assumed for a majority of the network layers. In addition, assuming biases to vary less is not unusual since there are relatively few of them and they have a strong influence on the model since they act on sums. The reason why the first fully connected layer is treated differently is that it covers much more parameters than the other layers. Indeed it includes 400,000 weights, while all the other layers together only contain 30,500 weights. Due to the large number of parameters in the first fully connected layer, we assume that the model will be more uncertain in the network parameters of this layer. So $\tau_{i}$ is initialized with 1 and $\tau_{bi}$ with 0.2.

Finally, all Bayesian models are optimized with the same mini-batch optimization procedure as their frequentist analogues. For computing the model accuracies, each test example is propagated 100 times through the network using Caffe’s bindings to Python. The test errors computed on the test dataset (N= 10,000 samples) and the absolute and relative decreases in the error with respect to the non-Bayesian models are given in Table 2. One can see that the Bayesian models always perform better than their frequentist analogues. For the first two models the accuracy is only slightly better, while the third model shows a significant improvement, especially if one considers the relative decrease of the test error. It is not surprising that the increase in accuracy is only small for the first two models since all models considered converge very well and do not suffer from overfitting because there is plenty of training data available. The third model which is trained using only 10,000 images shows signs of overfitting in the non-Bayesian case. The Bayesian network however is more robust towards overfitting and thus performs significantly better. This illustrates the advantage of Bayesian deep learning in the presence of only a limited number of training images.

**Figure 6:**
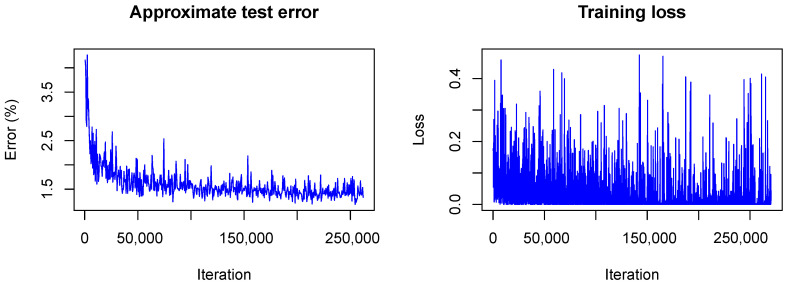
Visualization of the network training without dropout.

**Figure 7:**
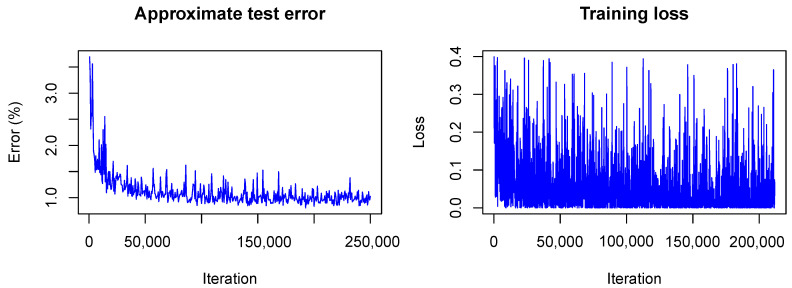
Visualization of the network training with dropout.

It is interesting to see how LeNet converges following our Bayesian approach. In Figure 6 and Figure 7 the training is visualized for the first and the second model, that is, the model trained without dropout and the model trained with dropout.

In contrast to the frequentist case, only the approximate test error is plotted. This means that only one sample of each testing image is used for predictions and that the weight scaling inference rule is applied. Currently, the Caffe framework does not provide other options for the testing phase during optimization. Nonetheless, the imprecise approximation of the test error gives a rough estimate of the real test error and therefore helps to understand what happens with the model accuracy during training. One can see that the loss *(plotted without the term due to the KL-divergence)* fluctuates heavily during training due to the random samples drawn from the variational distribution. However, the test error decreases quickly as in the non-Bayesian case and seems to keep decreasing as training goes on. This is not the case for the frequentist model (see  Figure 1) for which the test error seems to increase slowly. This, again, indicates the strength of our approach against overfitting.

**Figure 8:**
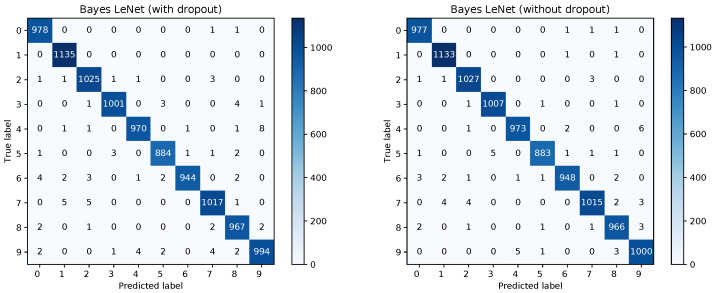
Confusion matrices for the Bayesian LeNet without and with dropout.

**Figure 9:**
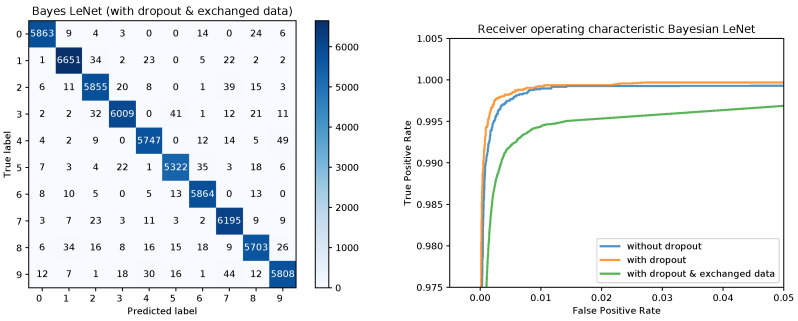
Confusion matrix for the Bayes LeNet with dropout and exchanged data and ROC curves for all three models.

Figure 8 shows the confusion matrices for the Bayesian LeNet without dropout (left) and with dropout (right). The confusion matrix for the Bayesian LeNet with dropout and exchanged training and testing data is shown in Figure 9 (left). In addition, the mean ROC curves for each of the Bayesian models is shown in Figure 9 (right). Comparing these results to the results of the frequentists networks from Figure 2 and Figure 3 confirms the superior performance of the Bayesian models, especially for the model with exchanged training and testing data where the initial slope for the Bayesian model is much steeper.

The a posteriori uncertainties are quite the same for all three Bayesian models. In Table 3, the uncertainties for the second model, that is, the model trained with dropout, are given. One can see that the model uncertainty is small for all layers except for the first fully connected one.

A value of 0.76 for $\tau_{3}$ indicates that the network is not even sure about the algebraic sign of the weights in this layer. Therefore, we assume that the network architecture is not optimal and reduce the number of output neurons for the first fully connected layer from 500 to 250. In the Bayesian case, this does not lead to a significant increase of the network accuracy but the network uncertainty for the first fully connected layer decreases significantly as one can see in Table 4.

This result indicates that the Bayesian approach can be used to optimize the model architecture both in terms of accuracy and model size for a given training and testing dataset. In this particular case, we were able to reduce the number of parameters by almost a factor of 2 while achieving the same accuracy. Even more interesting, when the reduced model is trained the classical way, the achieved accuracies become as good as for the Bayesian model, indicating again that the initial model was suffering from overfitting.

**Figure 10:**
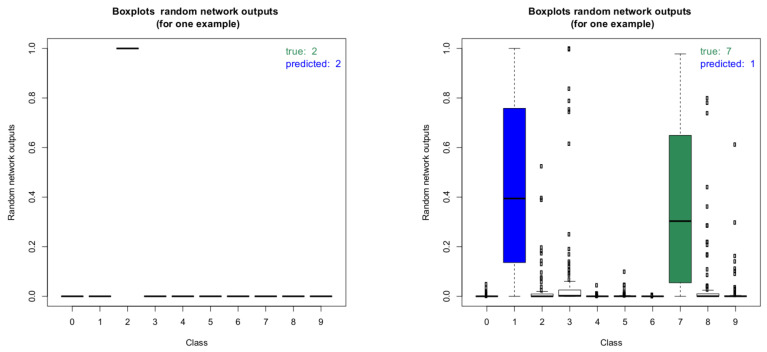
Boxplots of the random network outputs of two representative images of the MNIST dataset. Left: Boxplot of a correct classification, right: boxplot of an incorrect classification result.

In addition to providing information about the model uncertainty, our approach can also be used to determine the uncertainties of the predictions. Due to the random sampling of the weights and biases during each forward pass, accurate credible intervals can be estimated by performing multiple forward passes per image. This information can be used in applications using our algorithm for classification. For example, a check for statistical significance for the classification result can be performed and the result can be used to decide about the next steps in the application (e.g., proceed autonomously, repeat classification, escalate to user, etc.). Figure 10 shows two boxplots of the random network outputs (model without dropout) for two representative images from the MNIST test data set. On the left, the boxplot for an image with correct classification result is shown. Clearly, the network is very certain about this classification result. On the right, the boxplot for an image with wrong classification result is shown. As can be seen, the result for the wrongly predicted label is not statistically significant as there is a clear overlap between the boxes of the true label and the predicted one.

These boxplots were computed by performing the inference 100 times for each image. It is interesting to note that in the case of the wrongly predicted image on the right, the network produces very high outlier probabilities for other classes besides the true and the predicted label. This illustrates the potential for deterministic networks to produce wrong classifications with very high class probabilities. Checking for all images if the estimated 95% credible intervals of the predicted classes overlap with the 95% intervals of the other classes gives further insight into the prediction capabilities of the network. Table 5 summarizes the results for the model without dropout.

**Figure 11:**
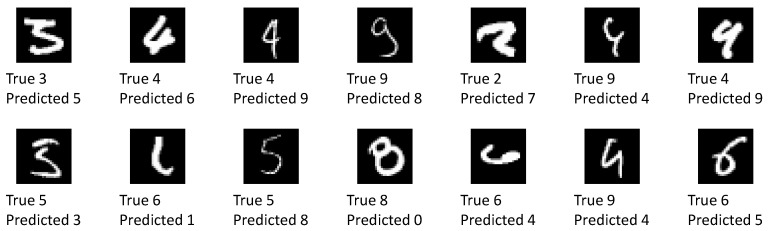
All 14 images where the network was sure within 95% credible intervals about its wrong classification result.

As can be seen, the overwhelming majority of classification results is correct and the network is also confident about these predictions. About 300 images are classified correctly, but the network is not sure within 95% credible intervals. A total of 94 images are classified incorrectly. In the vast majority of these cases, the network is unsure about the classification result. In only 14 cases, the network is quite sure about its wrong classification. Please note, that due to the random sampling of network parameters, the results are slightly different each time they are computed unless a very large number of forward passes is performed for each image. This is also the reason why the number of miss-classified images in this section differs from the one obtained above. The uncertainty analysis presented here was performed separately. From an application point of view, the latter case (quite certain about wrong results) is the most critical. Figure 11 shows all of the 14 images which have been classified wrongly with confidence by the network.

More than half of these images visually resemble the predicted label at least as much as they resemble the true label. The remaining images are without a doubt wrongly classified. Some of these images can be excluded by raising the confidence level requirement. A detailed investigation into wrong yet confident classification results is left for further study.

### 4.2. Comparison to Bayes by Backprop

In this section, we compare our approach to the ”Bayes by Backprop” algorithm by Blundell et al. [30]. As mentioned in Section 2.2, their approach also uses a normal distribution with diagonal covariance matrix as variational distribution but introduces one variance term for each expectation value. Their approach can thus be considered more flexible in capturing the true posterior distribution of the network parameters but comes at the cost of doubling the number of parameters to be optimized. As was pointed out before [12], this leads to convergence issues especially for larger network architectures. Thus, for our comparison, we repeated the same experiment presented in Reference [30] with our suggested approach: A small network consisting of only two hidden fully-connected layers with 1200 units and a rectified linear activation function each followed by a softmax output layer was trained on the MNIST dataset without data augmentation or dropout. The best results were obtained using SGD with mini-batches of 128 images, and a learning rate of 0.05 with momentum of 0.9. The initial values of the uncertainties of the layers have been set to 0.4 for $\tau_{i}$ and 0.1 for $\tau_{bi}$. The a priori variances are initialized to 2.0 for the biases and 1.0 for the weights. Table 6 summarizes the final test error of our approach along with the results obtained for Bayes by Backprop and a vanilla, frequentist SGD approach (both taken from Reference [30]).

As can be seen, our approach outperforms both the standard, frequentist SGD approach as well as the Bayes by Backprop approach with a Gaussian prior. Only the approach that combines Bayes by Backprop with a scale mixture prior achieves a lower test error. An investigation into whether our approach can also benefit from the combination with a scale mixture prior is subject to future work.

### 4.3. GoogLeNet and Custom Dataset

To illustrate that our approach is also applicable to large, modern network architectures with many more hidden layers and network parameters and also larger size input vectors, we trained a GoogLeNet architecture [43] on a custom dataset of 11 different classes of fruits and vegetables. This work was performed using the pytorch implementation (see Section 3.2). The dataset consists of a total of 2437 images, randomly split into a training dataset of 1952 images and a validation dataset of 485 images. The 11 classes of fruits an vegetables were apple, avocado, banana, blackberry, blueberry, carrot, cucumber, grape, peach, pear, and strawberry. The dataset was compiled from freely available online resources. The images were chosen such that only items belonging to one particular class were present in each image. No restrictions were placed on the background scenery provided it did not show other types of fruits and vegetables. The number of images in the training and test split for each class is shown in Table 7.

As the images are all of a different size, they are first randomly cropped and then resized to 224×224 pixels using the utility routine RandomResizedCrop provided by pytorch. Similarly to Section 4.1, we compare a frequentist model to our Bayesian approach. Both models are trained based on an Image-Net pre-trained version of GoogLeNet available from pytorch (https://download.pytorch.org/models/googlenet-1378be20.pth). In both cases, the pre-trained model is fine-tuned for 100 epochs using a learning rate of 0.01 with momentum of 0.9 and a batch size of 32. For the Bayesian approach, the a posteriori uncertainties are initialized the same for all layers. As before they take on the values of 0.4 for $\tau_{i}$ and 0.1 for $\tau_{bi}$. The a priori variances are initialized to 2.0 for the biases and 1.0 for the weights. The regularization term of the Kullback-Leibler divergence was weighted with a factor of $0.2\times 10^{-6}$. After 100 epochs, the frequentist model reaches an accuracy of 0.9 across the entire validation dataset. The Bayesian model reaches an accuracy of about 0.9 for a one-pass evaluation over the validation dataset (the exact number differs for each random forward pass). When averaging over the results of 100 forward passes for each image, the accuracy of the Bayesian model improves to 0.924.

**Figure 12:**
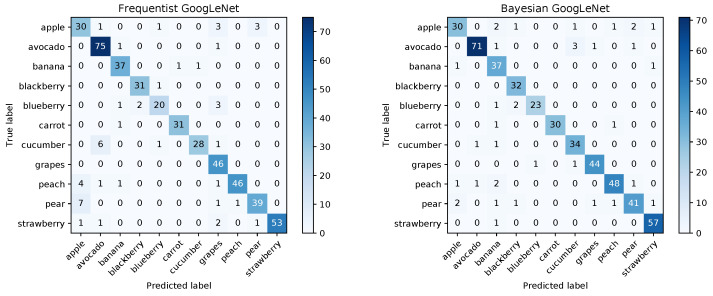
Confusion matrices for the frequentist and Bayesian GoogLeNet.

**Figure 13:**
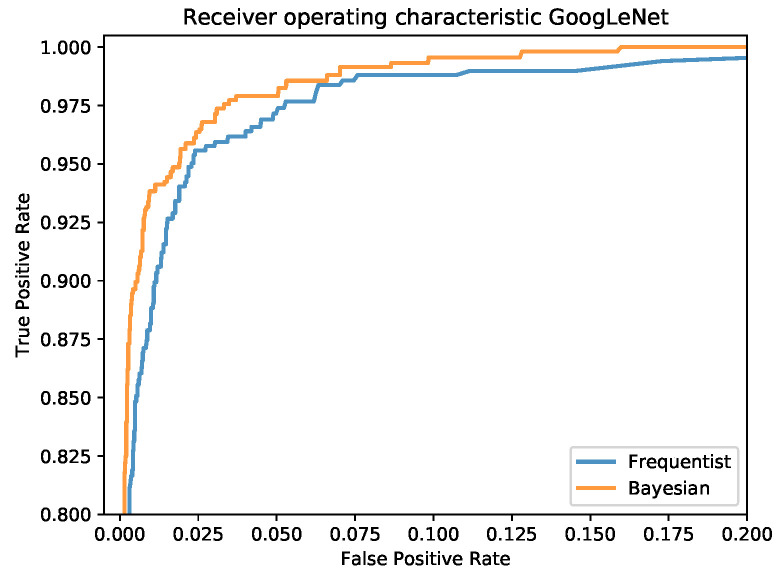
Mean ROC curves for the frequentist and the Bayesian model.

The confusion matrices for the frequentist and the Bayesian model are shown in Figure 12 and a comparison of the mean ROC curves obtained in Figure 13.

An analysis of the 95% credible intervals as before is summarized in Table 8.

As before, the analysis shows that the network is quite certain about the majority of its correct results and quite uncertain about all but one of its wrong results. This indicates that with the proper settings for the credible intervals, the vast majority of wrong classification results can be detected. Although it comes at the expense of a larger number of uncertain but correct results, this may be important for applications where sensitivity is of utmost importance. An investigation into how the prediction uncertainty depends on network architecture, training and testing datasets as well as network optimization hyperparameters is subject to ongoing work.

## 5. Conclusions

We present here a Bayesian approach to deep learning that allows for accurate calculation of the uncertainty of network predictions as well as the uncertainty of the model parameters while introducing only few additional parameters to be optimized. In particular, we introduce two variance terms per layer (one for the weight parameters, one for the biases) that are optimized during training along with the other network parameters. This makes our approach scalable to large, modern network architectures. Compared to classical, frequentist models, our approach is more robust against overfitting. Especially for small training datasets, a significant improvement in accuracy is obtained with our approach. In addition, information about network uncertainty can be readily interpreted and used for improvements of network architecture. Finally, our approach provides accurate uncertainty information about the predictions of the network with potentially significant impact for real-world applications.

## Figures and Tables

**Table 1:** Accuracies frequentist LeNet.

Model	Test Error
without dropout	0.93%
with dropout	0.75%
with dropout and exchanged data	1.94%

**Table 2:** Accuracies Bayesian LeNet.

	Test Error Reduction wrt. Frequentist Networks		
**Model**	**Test Error**	**Absolute**	**Relative**
without dropout	0.85%	0.08%	8.6%
with dropout	0.71%	0.04%	5.3%
dropout & exch. data	1.64%	0.3%	15.5%

**Table 3:** A posteriori uncertainty.

Layer	$\tau_{i}$	$\tau_{\mathrm{bi}}$
convolutional 1	0.003905073	0.01703058
convolutional 2	0.000045391	0.1021243
fully connected 1	0.7580626	0.1471348
fully connected 2	0.02509901	0.00004402

**Table 4:** A posteriori uncertainty reduced model.

Layer	$\tau_{i}$	$\tau_{\mathrm{bi}}$
convolutional 1	0.003570068	0.01361556
convolutional 2	0.000045395	0.1025237
fully connected 1	0.5672782	0.1359651
fully connected 2	0.01789308	0.00004324

**Table 5:** Summary of network performance (network without dropout).

	Quite Certain	Uncertain
correct	9609	297
wrong	14	80

**Table 6:** Summary of test errors.

Method	Test Error
Bayes by Backprop, Gaussian prior [30]	2.04%
Bayes by Backprop, Scale mixture prior [30]	1.32%
SGD (vanilla) [30]	1.88%
Ours	1.54%

**Table 7:** Train and validation images per class.

	Train	Test
Apple	155	38
Avocado	304	77
Banana	165	39
Blackberry	127	32
Blueberry	103	26
Carrot	129	32
Cucumber	143	36
Grape	185	46
Peach	217	53
Pear	187	48
Strawberry	237	58

**Table 8:** Summary of network performance (GoogLeNet).

	Quite Certain	Uncertain
correct	331	117
wrong	1	36

## References

[1] Krizhevsky A., Sutskever I., Hinton G.E. (2012). ImageNet Classification with Deep Convolutional Neural Networks.

[2] Bengio Y., Schwenk H., Senécal J.S., Morin F., Gauvain J.L. (2006). Neural Probabilistic Language Models. Innovations in Machine Learning.

[3] Hornik K., Stinchcombe M., White H. (1990). Universal Approximation of an Unknown Mapping and Its Derivatives Using Multilayer Feedforward Networks. Neural Netw..

[4] Liang S., Srikant R. Why Deep Neural Networks For Function Approximation?. Proceedings of the ICLR.

[5] Gulshan V., Peng L., Coram M., Stumpe M.C., Wu D., Narayanaswamy A., Venugopalan S., Widner K., Madams T., Cuadros J. (2016). Development and Validation of a Deep Learning Algorithm for Detection of Diabetic Retinopathy in Retinal Fundus Photographs. JAMA J. Am. Med. Assoc..

[6] Greenspan H., van Ginneken B., Summers R.M. (2016). Deep Learning in Medical Imaging: Overview and Future Promise of an Exciting New Technique. IEEE Trans. Med. Imaging.

[7] Li X., Ding Q., Sun J.Q. (2018). Remaining useful life estimation in prognostics using deep convolution neural networks. Reliab. Eng. Syst. Saf..

[8] Banerjee K., Dinh T.V., Levkova L. Velocity estimation from monocular video for automotive applications using convolutional neural networks. Proceedings of the IEEE Intelligent Vehicles Symposium.

[9] Jozwik K.M., Kriegeskorte N., Storrs K.R., Mur M. (2017). Deep Convolutional Neural Networks Outperform Feature-Based but not Categorical Models in Explaining Object Similarity Judgements. Front. Psychol..

[10] Heaton J., Polson N., Witte J. (2017). Deep learning for finance: Deep portfolios. Appl. Stoch. Model. Bus. Ind..

[11] Kendall A., Gal Y. (2017). What Uncertainties Do We Need in Bayesian Deep Learning for Computer Vision?. Advances in Neural Information Processing Systems 30.

[12] Gal Y., Ghahramani Z. Dropout as a Bayesian Approximation: Representing Model Uncertainty in Deep Learning. Proceedings of the 33rd ICML.

[13] Rowan A. (2017). Bayesian Deep Learning with Edward (and a Trick Using Dropout).

[14] Srivastava N., Hinton G., Krizhevsky A., Sutskever I., Salakhutdinov R. (2014). Dropout: A Simple Way to Prevent Neural Networks from Overfitting. J. Mach. Learn..

[15] Wan L., Zeiler M., Zhang S., LeCun Y., Fergus R. Regularization of Neural Networks Using DropConnect. Proceedings of the 30th ICML.

[16] Gal Y., Ghahramani Z. Bayesian Convolutional Neural Networks with Bernoulli Approximate Variational Inference. Proceedings of the ICLR.

[17] Kingma D.P., Salimans T., Welling M., Cortes C., Lawrence N.D., Lee D.D., Sugiyama M., Garnett R. (2015). Variational Dropout and the Local Reparameterization Trick. Advances in Neural Information Processing Systems 28.

[18] Goodfellow I., Bengio Y., Courville A. (2016). Deep Learning.

[19] Arora S., Bhaskara A., Ge R., Ma T. Provable Bounds for Learning Some Deep Representations. Proceedings of the 31st International Conference on Machine Learning.

[20] Polson N.G., Sokolov V. (2017). Deep Learning: A Bayesian Perspective. Bayesian Anal..

[21] Neal R.M. (1996). Bayesian Learning for Neural Networks.

[22] MacKay D.J.C. (1996). Bayesian Methods for Backpropagation Networks. Models of Neural Networks III.

[23] Toussaint U.V., Gori S., Dose V. (2006). Invariance priors for Bayesian feed-forward neural networks. Neural Netw..

[24] Lakshminarayanan B., Pritzel A., Blundell C., Guyon I., Luxburg U.V., Bengio S., Wallach H., Fergus R., Vishwanathan S., Garnett R. (2017). Simple and Scalable Predictive Uncertainty Estimation using Deep Ensembles. Advances in Neural Information Processing Systems 30.

[25] Hinton G.E., van Camp D. Keeping the neural networks simple by minimizing the description length of the weights. Proceedings of the Sixth Annual Conference on Computational Learning Theory.

[26] Jordan M.I., Ghahramani Z., Jaakkola T.S., Saul L.K. (1999). An Introduction to Variational Methods for Graphical Models. Mach. Learn..

[27] Blei D.M., Kucukelbir A., McAuliffe J.D. (2016). Variational Inference: A Review for Statisticians. arXiv.

[28] Bishop C.M. (2006). Pattern Recognition and Machine Learning.

[29] Gal Y., Ghahramani Z. (2016). Dropout as a Bayesian Approximation: Appendix. arXiv.

[30] Blundell C., Cornebise J., Kavukcuoglu K., Wierstra D. Weight Uncertainty in Neural Networks. Proceedings of the ICML.

[31] LeCun Y., Bottou L., Bengio Y., Haffner P. (1998). Gradient-Based Learning Applied to Document Recognition. Proc. IEEE.

[32] Louizos C., Welling M. (2016). Structured and Efficient Variational Deep Learning with Matrix Gaussian Posteriors. arXiv.

[33] Amari S.I. (1985). Differential-Geometrical Methods in Statistics.

[34] Hernandez-Lobato J.M., Li Y., Rowland M., Hernandez-Lobato D., Bui T., Turner R.E. (2015). Black-Box Alpha Divergence Minimization. arXiv.

[35] Li Y., Gal Y. (2017). Dropout Inference in Bayesian Neural Networks with Alpha-divergences. arXiv.

[36] Posch K., Pilz J. (2020). Correlated Parameters to Accurately Measure Uncertainty in Deep Neural Networks. IEEE Trans. Neural Netw. Learn. Syst..

[37] Kingma D.P., Welling M. (2013). Auto-Encoding Variational Bayes. arXiv.

[38] Hershey J.R., Olsen P.A. Approximating the Kullback Leibler Divergence Between Gaussian Mixture Models. Proceedings of the 2007 IEEE International Conference on Acoustics, Speech, and Signal Processing.

[39] Jia Y., Shelhamer E., Donahue J., Karayev S., Long J., Girshick R., Guadarrama S., Darrell T. (2014). Caffe: Convolutional Architecture for Fast Feature Embedding. arXiv.

[40] BVLC (2016). Caffe. https://github.com/BVLC/caffe.

[41] Paszke A., Gross S., Massa F., Lerer A., Bradbury J., Chanan G., Killeen T., Lin Z., Gimelshein N., Antiga L., Wallach H., Larochelle H., Beygelzimer A., Alché-Buc F., Fox E., Garnett R. (2019). PyTorch: An Imperative Style, High-Performance Deep Learning Library. Advances in Neural Information Processing Systems 32.

[42] LeCun Y., Cortes C., Burges C.J. The MNIST Database of Handwritten Digits.

[43] Szegedy C., Liu W., Jia Y., Sermanet P., Reed S., Anguelov D., Erhan D., Vanhoucke V., Rabinovich A. Going deeper with convolutions. Proceedings of the 2015 IEEE Conference on Computer Vision and Pattern Recognition.

[44] Bengio Y. (2012). Deep Learning of Representations for Unsupervised and Transfer Learning. JMLR.

